# Minimally-Invasive Conventional Right Colectomy Versus Complete Mesocolic Excision for Right-Colon Adenocarcinoma: A Single-Institution Cohort

**DOI:** 10.7759/cureus.95652

**Published:** 2025-10-29

**Authors:** Rodrigo Moisés de Almeida Leite, Lucas Pilotto Ramos, Ana Sarah Portilho, Francisco Tustumi, Lucas Cata Preta Stozlemburg, Lucas Soares Gerbasi, Lucas de Araújo Horcel, Rafael Vaz Pandini, Victor Edmond Seid, Sergio Araujo

**Affiliations:** 1 Colorectal Surgery, Einstein Hospital Israelita, São Paulo, BRA; 2 Gastrointestinal Surgery, Hospital da Clinicas da Falcudade de Medicina de Universidade de São Paulo (HCFMUSP), São Paulo, BRA; 3 Gastrointestinal Surgery, Universidade de São Paulo, São Paulo, BRA; 4 Surgical Gastroenterology, Einstein Hospital Israelita, São Paulo, BRA

**Keywords:** central vessel ligation, colon cancer, colorectal cancer, complete mesocolic excision, d3 lymphadenectomy, laparoscopic surgery, minimally invasive colectomy, right colon cancer, robotic surgery

## Abstract

Introduction: The benefits of complete mesocolic excision (CME) versus conventional right colectomy in right-sided colon cancer remain to be defined, as these relate to reduced nodal recurrence and increased disease-free survival (DFS). Optimal patient selection also appears crucial in selecting the best surgical technique for right colon cancer.

Methods: A prospective, single-center database analyzing minimally invasive CME versus conventional colectomy in right-sided colon cancer was retrospectively analyzed. Only patients with free surgical margins and without distant metastases were included for analysis. The outcomes analyzed comprised local recurrence, nodal recurrence, DFS, length of stay, node harvesting, and major postoperative complications. The analysis was adjusted for multiple confounders, including age, sex, BMI, pathological T stage, pathological N stage, mismatch repair protein deficiency, adjuvant chemotherapy, first postoperative carcinoembryonic antigen (CEA) level, and American Society of Anesthesiologists Physical Status Classification System (ASA) score.

Results: CME presented a similar safety profile, with increased lymph node yield. CME was associated with a significant reduction in the risk of nodal recurrence (adjusted relative risk (RR) = 0.08; 95% CI: 0.05 to 0.09; p < 0.001). Moreover, in the propensity score matching (PSM) analysis, CME was associated with a significant coefficient of reduction for nodal recurrence (Coef. = -0.14; 95% CI: -0.23 to -0.05; p = 0.01). An improvement in DFS was also observed (hazard ratio (HR) = 0.03; 95% CI: 0.003 to 0.27; p = 0.002) in patients with pT3/pT4 or pN+ disease.

Conclusion: Minimally invasive CME may be associated with decreased nodal recurrence and increased DFS in patients with right colon cancer.

## Introduction

Colorectal cancer is a leading cause of cancer-related morbidity and mortality worldwide. In right colon cancer, the concept of complete mesocolic excision (CME) has gained traction as a potentially superior surgical approach compared to conventional right colectomy. Introduced by Hohenberger et al. in 2009 [1], CME involves meticulous dissection along embryological planes to achieve an intact mesocolon with a higher yield of lymph nodes, which theoretically reduces the risk of local recurrence and improves disease-free survival (DFS) rates [2].

However, a recent systematic review and meta-analysis [3] failed to demonstrate significant improvement in three-year overall survival and DFS with the CME approach, and no differences were found in local or distant recurrence despite a significant increase in five-year overall survival. Several factors may affect this, such as an overall low incidence of local recurrence after curative resection of colon cancer and adjuvant chemotherapy [4].

Patient selection may be crucial in choosing CME as a technical approach. For more advanced cases of right colon cancer, that is, stages III/IV, a survival benefit has been more constant in observational trials [5]. This may be due to a relatively higher incidence - around 8% - of central node metastasis in T3 or T4 right colon tumors [6,7].

The safety of the CME procedure is also controversial. Due to the greater distance of the vascular tie and increased mesentery mobilization, this may be associated with greater intraoperative blood loss [8,9] and postoperative complications. However, a recent prospective study [10] has shown similar complication rates when the surgery is conducted in experienced centers.

To analyze this relevant clinical question, we conducted a retrospective analysis of a prospective database of a single center specializing in minimally invasive surgery for colon cancer, comparing the short-term safety and long-term survival of CME versus conventional colectomy for the management of right-sided colon cancer.

This article was previously posted to the Research Square preprint server on May 6, 2025.

## Materials and methods

Study design

A retrospective analysis was conducted of a database of patients who underwent elective surgery for right-sided colon cancer at Hospital Municipal Vila Santa Catarina (affiliated with Einstein Hospital Israelita), São Paulo, Brazil. All procedures were performed by one of the six experienced oncology surgeons at the academic center. No learning-curve cases were included for analysis.

The research was granted ethical approval by the Institutional Review Board (IRB) of Einstein Hospital Israelita, which exempted the requirement for informed consent (reference number: 68495923.6.0000.0071). In accordance with ethical standards, personal identifiers were anonymized. The study also adhered to the Brazilian General Data Protection Law (Lei Geral de Proteção de Dados (LGPD)) by taking appropriate protective measures. Anonymization techniques were employed to remove any information that could reveal the identities of the subjects.

Inclusion criteria

We included adult patients (aged >18 years) diagnosed with right colon adenocarcinoma. Only minimally invasive right colectomies (laparoscopic or robotic) were considered. The inclusion period comprised patients who underwent the primary operation for colon cancer between 2016 and 2022.

Exclusion criteria

Exclusion criteria included the presence of a second primary tumor, open surgery, and lack of follow-up information from medical charts. Additionally, for survival analysis, we included only patients with the following findings in the initial pathological staging for DFS comparison: pathological T3 or T4 tumor; any T status with nodal involvement (N1 or higher); surgical margins free of disease; and absence of metastatic disease on diagnosis.

Study groups and surgical procedures

Only minimally invasive cases performed via laparoscopic or robotic techniques were included.

The robotic technique involved the use of the da Vinci robot (Intuitive Surgical Inc., Sunnyvale, USA). A single docking with four robotic arms was positioned in a straight line from the right iliac region to the xiphoid, along with an auxiliary 12-mm laparoscopic port (Medtronic, Minneapolis, USA). The patient was positioned supine with mild left decubitus and in the Trendelenburg position.

In the case of the laparoscopic technique, five trocars (Medtronic) were placed for the procedure: one 11-mm trocar in the umbilicus for the optics, one 12-mm trocar in the left upper quadrant, and three 5-mm trocars in the remaining quadrants. The patient was positioned supine with mild left decubitus and in the Trendelenburg position. During the anastomosis, the patient was returned to a neutral position.

In both approaches, a laparoscopic advanced bipolar scalpel (Medtronic) and laparoscopic linear staples (Medtronic) were used in the procedure. For vessel ligation, only advanced bipolar energy was used, without clips. The anastomoses were lateral, ileal-colonic, and isoperistaltic, used 60-mm mid-width linear staplers (Medtronic), and concluded with barbed-wire sutures (Medtronic). The specimen was retrieved via Maylard auxiliary incision. 

We considered CME for a right colectomy with central ligation of the ileocolic, right colonic, and right branches of colon vessels, mesenteric dissection along the embryological layers, and D3 lymphadenectomy - including superior mesenteric vein (SMV) and lateral superior mesenteric artery (SMA) lymph nodes. Conventional colectomy included ligation of the ileocolic vessels in the SMV root after their exposure, without additional dissection of the D3 nodes.

Postoperative assessments and endpoints

Primary Outcome

Our primary outcome was locoregional recurrence, defined as nodal disease or local recurrence after follow-up for cancer was initiated. Nodal disease was considered positive if the imaging findings or pathological results indicated adenocarcinoma recurrence. All patients underwent postoperative surveillance according to the American Society of Clinical Oncology (ASCO) guidelines [11], including a clinical encounter with a clinical oncologist every three months, carcinoembryonic antigen (CEA) monitoring, and an annual CT scan of the chest, abdomen, and pelvis. DFS was defined as the absence of locoregional or distant recurrence after surgery.

Secondary Outcomes

The secondary outcomes were as follows: operative time, in-hospital stay, number of harvested lymph nodes, and postoperative surgical complications (including bleeding, unplanned conversion, deep surgical site infection, superficial surgical site infections, anastomotic leaks, and death) within 30 days.

Statistical analysis

We conducted survival analysis for DFS, counting in months from the operation date if no adjuvant chemotherapy was conducted or from the end of adjuvant chemotherapy to build Kaplan-Meier survival curves and obtain adjusted hazard ratios through Cox regression.

We also conducted multivariate Poisson regression to obtain risk ratios for locoregional recurrence across groups after adjusting for age, sex, BMI, pathological T stage, pathological N stage, mismatch repair protein deficiency, adjuvant chemotherapy, first postoperative CEA level, and American Society of Anesthesiologists Physical Status Classification System (ASA) score [12].

Additionally, we conducted propensity score matching using Stata nearest neighbor modeling, matching the cohorts by the same variables to analyze our primary outcome more homogeneously. We also conducted univariate and multivariate analyses adjusted for the same confounders for our secondary outcomes using Poisson regression. Due to the relevance of patient selection for CME, we also included a Bayesian variable inclusion regression map, visually highlighting the strongest predictors for nodal recurrence. 

All analyses were conducted using Stata 18 for Mac, Standard Edition (StataCorp LLC, College Station, USA).

## Results

Demographics

Overall, 154 cases were included for analysis, 26 in the CME cohort and 128 in the conventional cohort. After applying inclusion and exclusion criteria for the survival analysis, the cohort comprised 26 CME cases and 63 conventional colectomy cases.

The median age recorded was 65 years (IQR 60-76) in the conventional cohort and 65.5 years (IQR 55-70) in the CME cohort. Female patients comprised 54.69% of conventional colectomy cases and 53.85% of CME cases. No significant differences existed between the groups regarding T or N status or ASA scores. Patients also had similar baseline characteristics, such as preoperative hemoglobin and albumin levels. Table 1 summarizes the patient demographics.

**Table 1:** Demographic characteristics The p-values were obtained through t-tests for medians and proportion tests in Stata 18 software (StataCorp LLC, College Station, USA). CME: complete mesocolic excision; CEA: carcinoembryonic antigen; ASA: American Society of Anesthesiologists Physical Status Classification System

	CME, 26 (16.9%)	Conventional, 128 (83.12%)	p-value
Laparoscopic approach	20 (76.92%)	103 (80.47%)	0.327
Robotic approach	6 (23.07%)	25 (19.53%)	0.655
Median age (IQR)	65.5 years (55-70)	65 years (60-76)	0.097
Female patients	14 (53.85%)	70 (54.69%)	0.402
Male patients	12 (46.15%)	58 (45.3%)	0.372
Mismatch repair	5 (19.2%)	11 (8.59%)	0.334
pT1 or pT2	5 (19.2%)	19 (14.7%)	0.678
pT3 or pT4	21 (80.8%)	109 (85.3%)	0.66
ASA I or II	22 (84.6%)	109 (85.3%)	0.847
Preoperative Hb level (g/dL)	12.60 (SD 2.23)	12.11 (SD 1.99)	0.836
Preoperative albumin level (g/dL)	4.12 (SD 0.46)	4.26 (SD 0.40)	0.938
First postoperative CEA (ng/mL)	3.29 (SD 3.5)	3.37 (SD 2.84)	0.541

Locoregional recurrence

**Figure 1:**
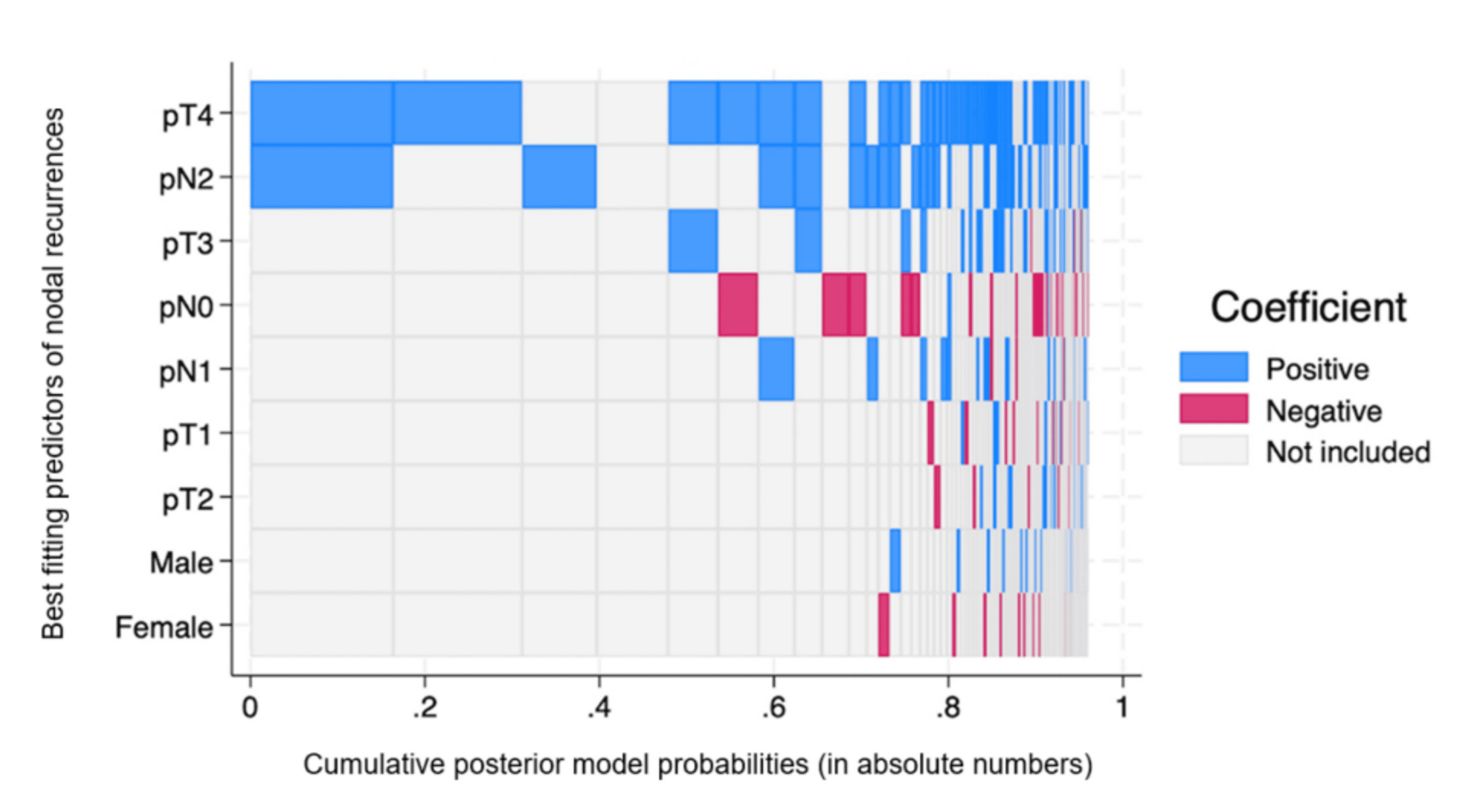
Bayesian regression variable selection map Only the top 100 models are shown out of 512 visited.

No local recurrences were observed in either group. Nodal recurrence was observed in six patients in the conventional cohort (incidence of 9.52%). No nodal recurrence was observed in the CME group. The mean time for nodal recurrence was 28 months (SD 10 months). The strongest predictors for nodal recurrence were pT4 and pN2 stages. Predictors for nodal recurrence are summarized in the Bayesian regression model variable inclusion map (Figure 1). In the baseline analysis, no difference existed in the prevalence of pT4 and pT3 tumors between the cohorts.

CME was associated with a significant decrease in nodal recurrence in the various models for analysis. 

After adjusting for multiple confounders using Poisson regression, CME was associated with a significant reduction in the risk of nodal recurrence (adjusted relative risk (RR) = 0.08; 95% CI: 0.05 to 0.09; p < 0.001). Moreover, in the propensity score matching (PSM) analysis and logistic regression model, CME was associated with a significant coefficient of reduction for nodal recurrence (Coef. = -0.14; 95% CI: -0.23 to -0.05; p = 0.01). Table 2 presents our post-PSM validation. The Bayesian regression variables inclusion map shows that pathological T3, T4, and N-positive patients were strongly associated with increased nodal recurrence.

**Table 2:** Postoperative and oncologic outcomes Adjusted RR and p-values were obtained through multivariate Poisson regression. HR and p-values were obtained through Cox regression. CME: complete mesocolic excision; RR: relative risk; HR: hazard ratio

	Conventional	CME	RR/HR	p-value
Postoperative outcomes				
Operative time (IQR)	135 min (110-170)	160 min (142-170)	-	0.139
In-hospital stay (IQR)	2 days (2-3)	3 days (2-5)	-	0.845
Postoperative complications	23.53%	18.75%	-	0.687
Harvested lymph nodes	31 (24-35)	39 (29-45)	-	0.05
Oncologic outcomes				
Local recurrence	0	0	-	-
Nodal recurrence	6	0	RR: 0.08 (0.05-0.09)	<0.001
Median surveillance time (months)	31.61 (SD 8.56)	38.4 (SD 5.36)	-	-
Disease recurrence	-	-	HR: 0.03 (0.003-0.27)	0.002

DFS

**Figure 2:**
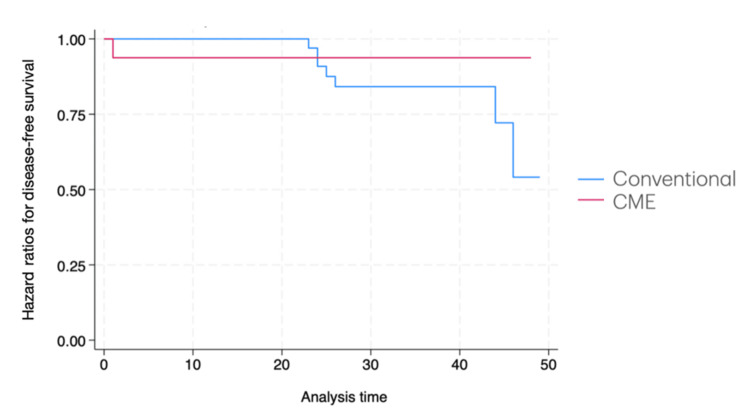
Kaplan-Meier survival estimates X-axis: time (months) from surgery or end of adjuvancy to recurrence. Y-axis: proportion of patients at risk for disease recurrence. CME: complete mesocolic excision

DFS was significantly increased in the CME cohort, as demonstrated in the Kaplan-Meier curve (Figure 2). The multivariate survival-time Cox regression showed a significant increase in DFS (hazard ratio (HR) = 0.03; 95% CI: 0.003 to 0.27; p = 0.002) for the CME-treated group. Only one death was recorded during the follow-up period, preventing the comparison of overall survival.

Postoperative outcomes

Operative Time

CME was associated with a significant increase in the total operative time. The median operative time was 135 minutes in the conventional cohort (IQR 110-170), compared to 162 minutes in the CME cohort (IQR 142-170). 

In-Hospital Stay

No significant difference existed between groups regarding in-hospital stays. The median in-hospital stay was three days in the CME cohort (IQR 2-5) and two days (IQR 2-3) in the conventional group. 

Harvested Lymph Nodes

CME was associated with a significant increase in harvested lymph nodes, with eight additional nodes on average. The median number of nodes was 31 in the conventional cohort (IQR 24-35) and 39 in the CME cohort (IQR 29-45). CME was not associated with positive pathological nodal disease (40% vs. 31%; RR = 1.28; 95% CI: 0.38 to 4.29; p = 0.692). 

Postoperative Complications

The incidence of major postoperative complications was 23.53% in the CME group and 18.75% in the conventional group. No significant difference existed in the incidence of major surgical complications in the univariate (RR = 1.31; 95% CI: 0.54 to 3.19; p = 0.539) and the multivariate regression (adjusted RR = 0.93; 95% CI: 0.35 to 2.46; p = 0.896). Only one case of major intraoperative bleeding was reported in the CME cohort, with an intraoperative blood transfusion required.

Table 2 summarizes the surgical outcomes.

## Discussion

In this retrospective cohort study, minimally invasive CME was associated with reduced nodal recurrence and increased DFS compared to minimally invasive conventional D2 right colectomy. The benefits were observed even after multiple adjustments and regression methods to reduce the risk of bias and confounding. These findings may translate to a survival benefit of this approach in clinical practice.

In our study, CME was associated with a higher number of harvested lymph nodes. The CME group had a median of 39 lymph nodes compared to 31 in the conventional colectomy group, yielding an average of eight additional nodes. This increased lymph node retrieval has been demonstrated in multiple studies comparing CME to conventional techniques, with around eight additional harvested nodes [9], and may be associated with more accurate staging, increased adjuvant chemotherapy, and reduced recurrence [10,13].

Our study showed a significant association of CME with reduced nodal recurrence. Nodal recurrence was observed in 9.52% of patients in the conventional cohort as opposed to zero events in the CME cohort. The mean time to nodal recurrence was 28 months. The strongest predictors for local recurrence were pT4 and pN2 stages, consistent with the literature. CME showed a consistent reduction in nodal recurrence risk across various models, including adjusted and PSM analyses.

Our analysis was also associated with a significant improvement in DFS for patients undergoing CME, as demonstrated in the Kaplan-Meier and Cox regression model. Patient selection may be crucial in this outcome. For our survival analysis, we only included patients with pT3, pT4, or pN+ disease. The recent Radical Extent of Lymphadenectomy of Laparoscopic Right Colectomy for Colon Cancer (RELARC) trial [14] failed to show a significant improvement in three-year DFS with CME, but the profile of patients differed with the inclusion of pT2N0 patients. Moreover, the location of tumors within the right colon (cecum vs. hepatic flexure tumors) [15] is also associated with differences in central node metastasis and may affect the findings. However, our findings correlate with a recent systematic review [9] showing an improved DFS with the same profile of patients as that included in our analysis. Also, our median node harvesting rate was adequate according to the ASCO guidelines [11] in both cohorts (>12), which assures the oncological quality of regular D2 colectomy. 

CME was associated with a longer operative time compared to conventional colectomy, with median times of 162 minutes for CME and 135 minutes for conventional surgery. This increase is expected, given the complexity of the CME procedure, which involves more extensive dissection and mesentery mobilization. Despite the longer operative time, the median in-hospital stay did not differ significantly between the two groups, indicating that the longer surgery duration does not necessarily lead to extended hospitalization or increased postoperative morbidity.

The incidence of major postoperative complications did not differ significantly between the CME and conventional groups. Major complications occurred in 23.1% of CME patients and 17.5% of conventional colectomy patients, with no statistically significant difference in univariate (RR = 1.31; 95% CI: 0.54 to 3.19; p = 0.539) or multivariate analyses (adjusted RR = 0.93; 95% CI: 0.35 to 2.46; p = 0.896). Notably, we recorded no superior mesenteric artery lesions or major vascular events. This shows the good safety profile of the CME procedure when conducted in specialized, high-volume centers.

However, our study has several limitations. Due to its observational and retrospective nature, the risk of residual bias cannot be excluded. Moreover, factors such as the decision to conduct CME initially may be associated with selection bias and inter-surgeon variation. Additionally, nodal recurrence had an overall low incidence (only six cases), which may impact model overfitting for our multivariate analysis. We also observed a trend toward a more robotic approach in the CME cohort, which may impact the results. The sample size was also small and may limit our findings. Finally, the overall low incidence of nodal recurrences limits the statistical significance of the findings due to the risk of model overfitting and an inability to conduct univariate analysis, as no nodal recurrences were observed in the CME cohort. However, we conducted multiple adjustments to reduce the influence of bias and systematic error, and all procedures were conducted by the same team of specialized surgeons in a high-volume academic center for colon cancer treatment.

## Conclusions

Our study demonstrates that CME is a safe and feasible surgical approach for right-sided colon adenocarcinoma. Compared with conventional colectomy, CME was associated with an increased lymph node yield without adding significant morbidity, and postoperative recovery remained comparable. Importantly, the oncological benefits of this technique appear most pronounced in carefully selected high-risk subgroups, particularly patients with locally advanced tumors (T3-T4) and/or node-positive disease in the absence of synchronous distant metastasis. By ensuring an intact mesocolic plane dissection and central vascular ligation, CME not only reduces nodal recurrence but also contributes to improved DFS, underscoring its potential to alter the natural history of stage II-III colon cancer. While our results are encouraging, they highlight the necessity of multicenter validation and prospective randomized trials to confirm reproducibility and generalizability. Furthermore, refinement in patient selection criteria, coupled with standardized operative protocols, may help consolidate the role of CME as a preferred surgical option in high-risk localized disease.

From a technical perspective, CME requires advanced familiarity with embryologic planes and precise identification of vascular anatomy, demanding a higher level of surgical expertise and structured training. Our findings suggest that when performed by adequately trained colorectal surgeons, CME provides not only superior nodal clearance but also tangible improvements in DFS for biologically aggressive tumors, especially locally advanced ones. These data support the consideration of CME as a reference technique for right-sided colon cancer in tertiary centers and specialized units, and they provide a rationale for its inclusion in surgical training curricula and future guideline updates.
